# Chronic kidney disease and poor outcomes in ischemic stroke: is impaired cerebral autoregulation the missing link?

**DOI:** 10.1186/s12883-018-1025-4

**Published:** 2018-03-02

**Authors:** Pedro Castro, Elsa Azevedo, Isabel Rocha, Farzaneh Sorond, Jorge M. Serrador

**Affiliations:** 1 Department Neurology, São João Hospital Center, Faculty of Medicine of University of Porto, Alameda Professor Hernani Monteiro, 4200-319 Porto, Portugal; 2 Cardiovascular Autonomic Function Lab, Institute of Physiology, Faculty of Medicine of University of Lisbon, Lisbon, Portugal; 3 Department of Neurology, Division of Stroke and Neurocritical, Northwestern University Feinberg School of Medicine, Chicago, IL USA; 4 Department of Pharmacology, Physiology and Neuroscience, Rutgers Biomedical Health Sciences, Newark, NJ USA; 5 Veterans Biomedical Research Institute and War Related Illness and Injury Study Center, Department of Veterans Affairs, East Orange, USA; 6 Cardiovascular Electronics, National University of Ireland Galway, Galway, Ireland

**Keywords:** Cardiovascular disease, Chronic kidney disease, Glomerular filtration rate, Stroke; transcranial Doppler

## Abstract

**Background:**

Chronic kidney disease increases stroke incidence and severity but the mechanisms behind this cerebro-renal interaction are mostly unexplored. Since both vascular beds share similar features, microvascular dysfunction could be the possible missing link. Therefore, we examined the relationship between renal function and cerebral autoregulation in the early hours post ischemia and its impact on outcome.

**Methods:**

We enrolled 46 ischemic strokes (middle cerebral artery). Dynamic cerebral autoregulation was assessed by transfer function (coherence, phase and gain) of spontaneous blood pressure oscillations to blood flow velocity within 6 h from symptom-onset. Estimated glomerular filtration rate (eGFR) was calculated. Hemorrhagic transformation (HT) and white matter lesions (WML) were collected from computed tomography performed at presentation and 24 h. Outcome was evaluated with modified Rankin Scale at 3 months.

**Results:**

High gain (less effective autoregulation) was correlated with lower eGFR irrespective of infarct side (*p* < 0.05). Both lower eGFR and higher gain correlated with WML grade (*p* < 0.05). Lower eGFR and increased gain, alone and in combination, progressively reduced the odds of a good functional outcome [ipsilateral OR = 4.39 (CI95% 3.15–25.6), *p* = 0.019; contralateral OR = 8.15 (CI95% 4.15–15.6), *p* = 0.002] and increased risk of HT [ipsilateral OR = 3.48 (CI95% 0.60–24.0), *p* = 0.132; contralateral OR = 6.43 (CI95% 1.40–32.1), *p* = 0.034].

**Conclusions:**

Lower renal function correlates with less effective dynamic cerebral autoregulation in acute ischemic stroke, both predicting a bad outcome. The evaluation of serum biomarkers of renal dysfunction could have interest in the future for assessing cerebral microvascular risk and relationship with stroke complications.

**Electronic supplementary material:**

The online version of this article (10.1186/s12883-018-1025-4) contains supplementary material, which is available to authorized users.

## Background

Chronic kidney disease (CKD) increases the risk of ischemic stroke [1, 2], its severity and the chance of poor outcome [3, 4]. The exact mechanisms that govern this cerebro-renal interaction are poorly understood [5]. A possible explanation may involve the similar features of kidney and brain microvascular beds. Both vascular beds are low resistance arterial beds that rely on continuous blood flow that ismaintained at relatively constant levels by a fine-tuned myogenic regulatory system. This confers protection to both the brain and kidney from arterial blood pressure (ABP) fluctuations that could cause large swings in blood flow [6–8]. This property, called autoregulation, has been long known to exist in renal [9] and cerebral [10, 11] vasculatures. Despite differences between organ systems, autoregulation is viewed as a generalized vascular protective mechanism [8] witch failure results in microvessel damage (e.g. from uncontrolled hypertension and diabetes [12–15]). In fact, glomerular sclerosis of chronic kidney failure [16], as well as lacunar infarcts and white matter lesions (WML) in the brain [12, 17], are all characterized by similar pathological conditions: endothelial dysfunction; ischaemic arteriosclerosis; low perfusion and small vessel leakage [12, 17]. Recent evidence shows that kidney impairment is associated with a greater severity of cerebral WML [18].

Because microvascular dysfunction impairs myogenic autoregulation, this could be the missing link between renal dysfunction and stroke morbidity and worthwhile of being explored. Dynamic cerebral autoregulation (CA) is rapidly and noninvasively assessed at the patient bedside by transfer function analysis (TFA) using spontaneous oscillations in ABP and cerebral blood flow velocity (CBFV) [14, 19–22] which is known to be impaired in acute ischemic stroke [23].

In this study, we examined the relationship between renal function and CA, assessed from TFA measured within 6 h of symptom-onset and its impact on long-term outcome.

## Methods

### Population studied

The study was conducted in Hospital Center São João, Porto, and its local ethical committee approved the study and all participants or proxy signed written informed consent. We included consecutive patients with acute ischemic stroke admitted to our stroke unit and with time from symptom onset, or from last seen well, within 6 h. Most patients admitted after 8:00 p.m. or weekends were not enrolled because transcranial Doppler (TCD) monitoring was not available. Exclusion criteria included hemodynamic instability requiring vasoactive agents, other central neurological co-morbidities, acute renal injury [24] or insufficient temporal window. We recruited 46 patients with ischemic stroke in middle cerebral artery (MCA) territory. All participants underwent cervical and transcranial ultrasound studies (Vivid e; GE) before evaluation to exclude hemodynamically significant extra- (> 50% in carotid artery calculated by NASCET method) or intracranial stenosis (≥ 50% by angle-corrected velocimetric criteria). Blood samples were collected at admission. We calculated Estimated glomerular filtration rate (eGFR) using the Chronic Kidney Disease Epidemiology Collaboration formula [25–27]. In the first fasting sample, we obtained total, low-density lipoprotein (LDL) and high-density lipoprotein (HDL) cholesterol, glucose and glycated hemoglobin (HgA1C).

### Clinical assessment

All patients underwent a neurological examination at presentation and National Institute of Health Stroke Scale (NIHSS) scores were calculated at baseline. The outcome, assessed by clinical interview, corresponded to mortality and functional independence [modified Rankin Scale (mRS) 0–2] at 90 days.

### Monitoring protocol

Evaluations were carried out in the stroke unit with the head of the bed at 0°. ABP was continuously monitored with a finger cuff in the unaffected side using Finometer MIDI (FMS, Amsterdam, Netherlands). Additionally, ABP was assessed with oscillometric cuff (Dash 2500, GE, UK). Heart rate was assessed from lead II of a standard 3-lead electrocardiogram. CBFV was recorded bilaterally from M1 segment of MCA (depth of 50–55 mm) with 2-MHz monitoring probes secured with a headband (Doppler BoxX, DWL, Singen, Germany). End-tidal carbon dioxide was evaluated by nasal cannula attached to Respsense capnograph (Nonin, Amsterdam, The Netherlands). All data was synchronized at 400 Hz with Powerlab (AD Instruments, Oxford, UK) and stored for offline analysis. Data collection occurred for 10 min within 6 h from symptoms-onset.

### Cerebral autoregulation data analysis

All signals were inspected using a custom based Matlab® program and artifacts removed by linear interpolation [28]. Systolic, mean and diastolic values of ABP and CBFV were calculated. For each heart-beat estimated cerebrovascular resistance (CVRi) was calculated by mean ABP/CBFV, reflecting vasomotor function [29]. TFA was used to assess dynamic CA by calculating coherence, gain and phase parameters from 10 min of beat-to-beat spontaneous oscillations of CBFV and ABP as previously reported [26, 27, 30, 31]. Coherence, gain and phase are reported for the low-frequency range (LF: 0.03–0.15 Hz), which is the autoregulatory frequency [6, 20, 21, 30], and also at high-frequency (HF: 0.15–0.5 Hz) band. In short, coherence is the coefficient of correlation between the signals; higher coherence between the oscillations is reflective of less effective CA. Gain quantifies the damping effect of CA on the magnitude of ABP oscillations. Phase shift represents the time delay between ABP and CBFV oscillations. Lower gain and higher phase represent tighter, more effective autoregulatory response [20].

#### Neuroimaging assessment

Computed Tomography brain scan (Siemens Somaton Emotion Duo, Erlangen, Germany), with 3 to 6 mm slices, was performed at presentation and repeated at 24 h. Hemorrhagic transformation was defined as any hemorrhage ranging from petechial hyperdensities to parenchymal hematoma [32]. WML, a correlate of cerebral microvascular disease, [17] were graded by the van Swieten [33] scale based on the first scan, a method validated against MRI in ischemic stroke [34].

### Statistical analysis

Normality was determined by Shapiro-Wilk test. Baseline data are presented for all subjects. The relationship between baseline characteristics, including eGFR, and CA parameters were determined by multivariate and univariate linear regression analysis adjusting for age, gender and other significantly related variables at previous univariate analysis. To better depict the relationship between CA and eGFR, we divided the first by median and the latter into subgroups of low and high (< 60 and ≥60 mL/min/1.73 m^2^, respectively) subgroups. Any subgroups were compared with Kruskal-Wallis and Mann-Whitney tests. The impact of eGFR on the outcome (mRS 0–6) and association with WML grades (0–4) were characterized by ordinal regression analysis. The risk of hemorrhagic transformation was assessed by logistic regression analysis. To avoid spurious associations, we considered statistically significant at *P* < 0.01 level for univariate regression analysis. Otherwise, *P* < 0.05 cut-off was used. All statistics were performed using IBM Statistical Package for Social Sciences (SPSS) Statistics v21™.

## Results

### Renal function and cerebral autoregulation

**Fig. 1:**
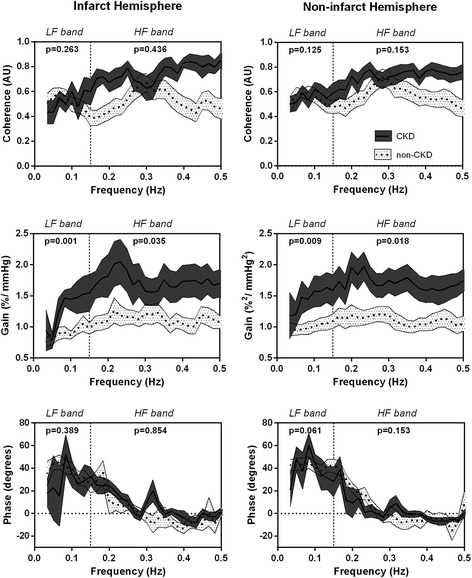
Frequency spectra from 0.03 to 0.5 Hz of Coherence and Transfer Function Gain and Phase measured in acute stroke patients (< 6 h) accordingly to their estimated glomerular filtration rate (eGFR). Continuous and spotted lines within dark grey and spotted white strips corresponds to means±SE of groups of low (< 60 mL/min/1.73 m^2^) and high (≥60 mL/min/1.73 m^2^) subgroups of eGFR, respectively. Groups were compared with Mann-Whitney and *P* values presented at right superior corner of each plot. Lower GFR shows higher gain values bilaterally which indicate less effective dynamic cerebral autoregulation

Baseline characteristics and hemodynamic data are reported in Tables 1 and 2, respectively. In a multivariate linear regression analysis, greater LF and HF gain values (indicating less effective CA) within 6 h of symptoms were significantly associated with lower eGFR irrespective of infarct side (*p* < 0.05, Table 3). Phase and coherence were not related to any baseline variable. Additional file 1: Table S4 shows the results of univariate linear regression. Figure 1 shows the distribution of CA parameters throughout all spectrum of frequencies. We found significantly higher gain values in low eGFR subgroup compared to the high one (*p* < 0.05).

**Table 1:** Demographics and baseline characteristics of patients (*N* = 46)

*Demographics*	
Male, n (%)	19 (41)
Age, years (mean ± SD)	73 ± 12
BMI, Kg.m^− 2^ (mean ± SD)	27 ± 5
Previous stroke/TIA, n (%)	6 (13)
Hypertension, n (%)	28 (61)
Diabetes Mellitus, n (%)	14 (30)
Dyslipidemia, n (%)	27 (59)
Tobacco, n (%)	4 (9)
Previous MI, n (%)	2 (4)
Atrial Fibrillation, n (%)	20 (43)
*Chronic Medication, n (%)*	
Antiplatelet	18 (39)
Statin	17 (37)
Beta-Blocker	10 (22)
ACEI/ARB	15 (33)
CCB	9 (20)
*Stroke characteristics*	
Etiology (TOAST), n (%)	
Large Vessel	6 (13)
Cardioembolic	18 (39)
Small vessel	2 (4)
Other	1 (2)
Undetermined	12 (26)
Occlusion of affected MCA	16 (35)
Baseline NIHSS score *[median(IQR)]*	14 (9–22)
*Neuroimaging [median(IQR)]*	
Infarct Volume, mL	19 (2–139)
Leucoencephalopathy grade (VanSwieten scale [33])	2 (1–3)
*Laboratorial (mean ± SD)*	
Total cholesterol, mg/dL	174 ± 72
LDL cholesterol, mg/dL	105 ± 42
HDL cholesterol, mg/dL	46 ± 11
Triglycerides, mg/dL	111 ± 68
Glucose, mg/dL	140 ± 81
HbA1C, %	6.4 ± 1.4
Creatinine, mg/dL	1.0 ± 1.0
*Renal Function*	
eGFR, ml/min/1.73 m^2^	80 ± 39
CDK–EPI Stage 0–1, n (%)	10 (22)
CDK–EPI Stage 2, n (%)	23 (50)
CDK–EPI Stage 3, n (%)	13 (28)
CDK–EPI Stage 4, n (%)	1 (2)
CDK–EPI Stage 5, n (%)	1 (2)

Body-Mass Index (*BMI*), Transitory Ischemic Attack (*TIA*), angiotensin-conversion-enzyme inhibitor (*ACEI*), angiotensin receptor blocker (*ARB*), calcium channel blocker (*CCB*), Trial of ORG10172 in Acute Stroke Treatment (*TOAST*), middle cerebral artery (*MCA*), National Institutes of Health Stroke Scale (*NIHSS*), Low-density lipoprotein (*LDL*), High-density lipoprotein (*LDL*), glycated hemoglobin (*HbA1C*), eGFR (estimated glomerular filtration rate) by Chronic Kidney Disease Epidemiology Collaboration (*CKD-EPI*) formula [25]

**Table 2:** Systemic and cerebral hemodynamic characteristics of patients (*N* = 46)

*Systemic hemodynamics (mean ± SD)*	
Systolic ABP, mmHg	144 ± 19
Mean ABP, mmHg	98 ± 11
Diastolic ABP, mmHg	75 ± 11
Heart rate, bpm	67 ± 11
LF mean ABP variability, mmHg^2^	85 ± 111
HF mean ABP variability, mmHg^2^	13 ± 20
EtCO_2_, mmHg	36 ± 7
*Cerebral hemodynamics (mean ± SD)*	
Ipsilateral	
MFV, cm/s	40 ± 20
CVRi, mmHg/(cm/s)	2.3 ± 0.9
LF MFV variability, (cm/s)^2^	128 ± 142
HF MFV variability, (cm/s)^2^	35 ± 49
Contralateral	
MFV, cm/s	48 ± 17
CVRi, mm Hg/(cm/s)	1.7 ± 0.7
LF MFV variability, (cm/s)^2^	200 ± 250
HF MFV variability, (cm/s)^2^	47 ± 68
Dynamic cerebral autoregulation	
Ipsilateral	
LF coherence, a.u.	0.5 ± 0.2
LF gain, %^2^/mmHg^2^	1.0 ± 0.3
LF phase, degrees	32 ± 23
HF coherence, a.u.	0.6 ± 0.2
HF gain, %^2^/mmHg^2^	1.5 ± 0.6
HF phase, degrees	– 0.1 ± 13
Contralateral	
LF coherence, a.u.	0.5 ± 0.2
LF gain, %^2^/mmHg^2^	1.2 ± 0.5
LF phase, degrees	44 ± 26
HF coherence, a.u.	0.7 ± 0.2
HF gain, %^2^/mmHg^2^	1.5 ± 0.6
HF phase, degrees	– 0.7 ± 13

Arterial blood pressure (*ABP*), mean flow velocity (*MFV*), estimated cerebrovascular resistance (*CVRi*), end-tidal carbon dioxide (*EtCO2*), Low (LF: 0.3–0.15 Hz) and high (HF: 0.15–0.5 Hz) frequency spectral bands; a.u., arbitrary units

**Table 3:** Association between estimated glomerular filtration rate and cerebral autoregulation parameters in acute stroke (< 6 h)

	Multiple Linear Regression – Beta coefficients and estimated 95% confidence interval					
	Coherence (a.u.)		Gain (%^2^/ mmHg^2^)		Phase (degrees)	
	LF band	HF band	LF band	HF band	LF band	HF band
*Ipsilateral (N = 37)*						
eGFR, ml/min	– 0.01 (− 0.01, 0.01)	– 0.08 (− 0.23, 0,15)	*– 0.01 (− 0.01,–0.004)	*– 0.02 (− 0.18,–0.003)	0.10 (− 0.24, 0.43)	– 0.08 (− 0.26, 0.14)
CCB Use	– 0.32 (− 10.6, 0.75)	– 0.12 (− 10.6, 0.75)	* 0.52 (0.22, 0.71)	* 0.35 (0.06, 1.12)	0.17 (− 0.09, 0.26)	0.17 (− 0.05, 0.12)
Mean ABP, mmHg	0.01 (− 0.01, 0.01)	0.06 (− 0.27, 0.37)	– 0.39 (− 0.02, 0.001)	– 0.01 (− 0.03, 0.01)	0.28 (− 1.21, 0.60)	0.28 (− 1.21, 0.60)
Heart Rate, bpm	– 0.11 (− 0.01, 0.00)	– 0.02 (− 0.43, 0.37)	– 0.30 (− 0.03, 0.03)	– 0.32 (− 0.01, 0.02)	0.13 (− 0.33, 1.26)	0.24 (− 1.23, 0.23)
EtCO_2_, mmHg	– 0.13 (− 0.01, 0.01)	– 0.03 (− 0.78, 0.67)	– 0.34 (− 0.08, 0.05)	– 0.23 (− 0.10, 0.02)	0.31 (− 0.10, .34)	0.13 (− 0.13, 2.31)
*Contralateral (N = 46)*						
eGFR, ml/min	– 0.19 (−0.01, 0.01)	0.09 (− 0.13, 0.25)	*– 0.01 (− 0.01,–0.004)	*– 0.02 (− 0.02,–0.004)	0.06 (− 0.23, 0.35)	0.09 (− 0.13, 0.25)
CCB Use	– 0.15 (− 34.5, 10.2)	– 0.32 (− 45.6, 7.5)	* 0.40 (0.16, 0.81)	0.25 (− 0.04, 0.76)	0.14 (− 0.06, 0.24)	0.17 (− 0.05, 0.12)
Mean ABP, mmHg	– 0.20 (− 0.01, 0.001)	0.10 (− 0.22, 0.45)	*– 0.02 (− 0.03,–0.01)	*– 0.02 (− 0.03, − 0.01)	0.17 (− 0.26, 0.84)	0.21 (− 1.23, 1.01)
Heart Rate, bpm	– 0.08 (− 0.01, 0.003)	– 0.02 (− 0.45, 0.38)	– 0.15 (− 0.02, 0.01)	– 0.32 (− 0.01, 0.13)	– 0.04 (− 0.58, 1.03)	– 0.10 (− 2.33, 0.12)
EtCO_2_, mmHg	– 0.03 (− 0.01, 0.01)	– 0.17 (− 1.12, 0.28)	– 0.29 (− 0.04, 0.00)	– 0.24 (− 0.01, 0.12)	0.13 (0.14, 2.56)	– 0.25 (− 0.23, 3.12)

Estimated glomerular filtration rate (eGFR), arterial blood pressure (MBP), end-tidal carbon dioxide (EtCO_2_), low (LF: 0.03–0.15 Hz) and high (HF: 0.15–0.5 Hz) frequency spectral bands, a.u., arbitrary units

*P < 0.01 for multivariate linear regression analysis adjusting for age, gender and all significantly related variables in univariate analysis (Additional file 1: Table S4)

**Fig. 2:**
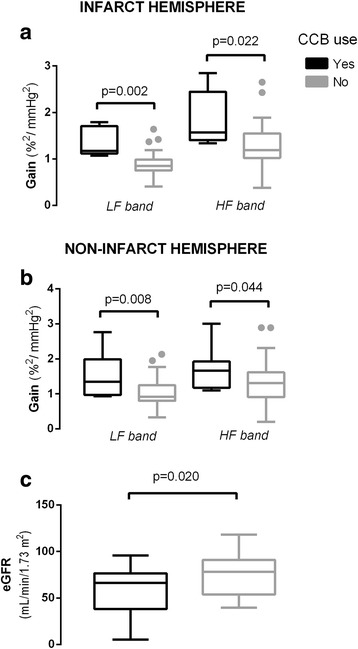
Box plots comparing median and quartiles distribution of cerebral autoregulation. Gain, at low (LF: 0.03–0.15 Hz) and high (HF: 0.15–0.5 Hz) frequency bands (subpanels A and B), and estimated glomerular filtration rate (GFR, subpanel C) by groups with and without chronic use of calcium channel blockers (CCB). Statistical *P* value of Mann-whitey test (**a**, **b**) and T-test (**c**) is presented

Previous use of Calcium Channel Blockers (CCB) was associated with higher gain values bilaterally (*p* < 0.05, Table 3). To explore further the influence of chronic medication with CCB we compared gain values in those using CCB (*n* = 10) compared to patients who were not on CCB (Fig. 2a and b) as well as renal function (Fig. 2c). CCB use was associated with higher gain values (*p* < 0.05) and also lower eGFR (57 and 75 ml/min, *p* = 0.022) but this was not statistically significant if we only compared chronically hypertensive patients (*N* = 37, 57 and 70 ml/min, *p* = 0.082). ABP at presentation was not different between those on CCB compared to those not on CCB (systolic 137 ± 21 vs 138 ± 20 mmHg, *p* = 0.901; diastolic 47 ± 12 vs 55 ± 14 mmHg, *p* = 0.125).

#### Relationship with cerebral microvascular disease (WML)

**Fig. 3:**
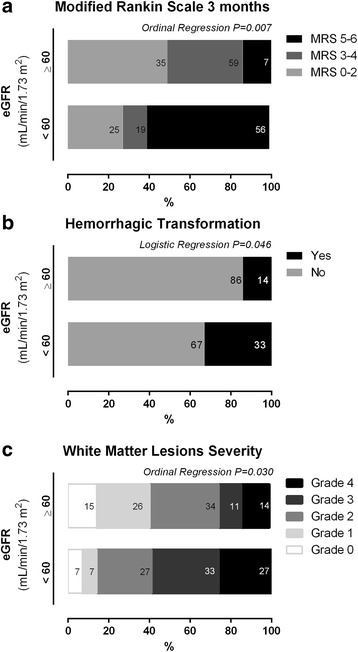
Relationship between renal function subgroups of low (< 60 mL/min/1.73 m^2^) and high (≥60 mL/min/1.73 m^2^) subgroups of estimated glomerular filtration rate (eGFR) and outcome accordingly to modified Rankin scale (**a**), risk of hemorrhage (**b**) and the severity of white matter lesions assessed at 24-h head Computed Tomography, accordingly to vanSwieten scale [33]. Subgroups were compared with ordinal regression or logistic regression as appropriate

**Fig. 4:**
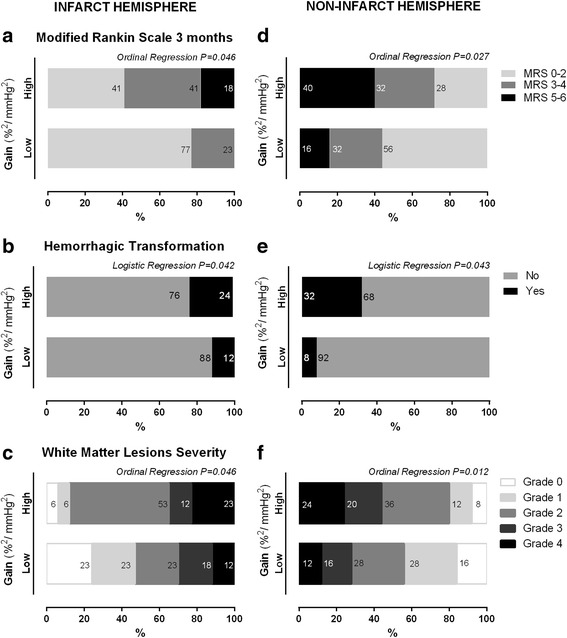
Relationship between cerebral autoregulation within 6 h of symptoms by subgroups of low and high LF gain subgroups for infarct (≤0.90, > 0.9%^2^/mmHg^2^, respectively) and non-infarct hemisphere (≤1.10, > 1.10%^2^/mmHg^2^, respectively) and outcome accordingly to modified Rankin scale (**a**), risk of hemorrhage (**b**) and the severity of white matter lesions assessed at 24-h head Computed Tomography, accordingly to vanSwieten scale [33]. Subgroups were compared with ordinal regression or logistic regression as appropriate

**Fig. 5:**
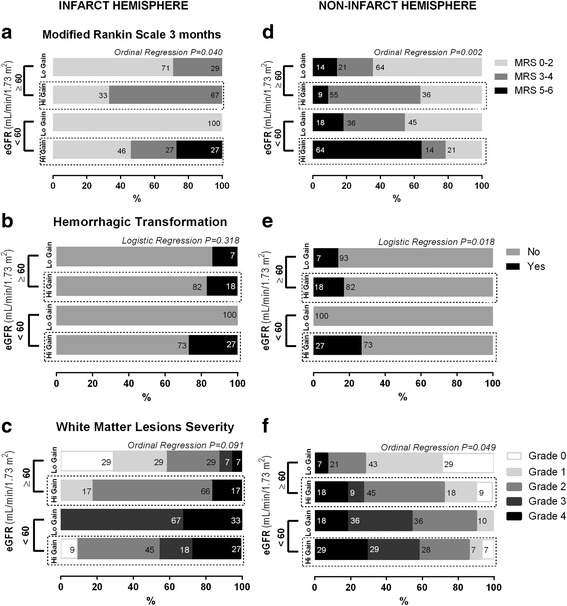
Interaction between cerebral autoregulation and renal function on outcome accordingly to modified Rankin scale (**a**), risk of hemorrhage (**b**) and on severity of white matter lesions assessed at 24-h head Computed Tomography, accordingly to vanSwieten scale [33]. Subgroups were created by splitting renal function into low (< 60 mL/min/1.73 m^2^) and high (≥60 mL/min/1.73 m^2^) estimated glomerular filtration rate (eGFR); cerebral autoregulation into low (Lo gain) and high (Hi gain) subgroups of LF gain in infarct (≤ 0.90 and > 0.90%^2^/mmHg^2^) and non-infarct hemisphere (≤ 1.10 and > 1.10%^2^/mmHg^2^). Notice that higher gain values represents worse levels of cerebral autoregulation. The interaction effect in outcome and white matter lesions was tested in multinomial logistic or ordinal regression models as appropriate

WML severity was correlated in an inverse manner with lower levels of eGFR (rho Spearman = − 0.52, *p* < 0.0001) and proportionally to higher levels of LF gain (ipsilateral *r* = 0.368, *p* = 0.040; contralateral *r* = 0.402, *p* = 0.006). This can be depicted clearly in figs. 3c and 4c+F, where we explored the distribution of WML grades accordingly to subgroups of low and high eGFR and LF gain, respectively. In Fig. 5c and f, we demonstrate the interaction between renal function and dynamic CA, by studying subgroups of eGFR versus subgroups of LF gain. Lower eGFR and higher LF gain (worse CA) were associated with more severe white matter lesion grades (ordinal shift analysis of infarct side *p* = 0.045 and non-infarct side *p* = 0.009).

### Renal function, cerebral autoregulation and outcome

As presented in Fig. 4a, lower GFR reduces significantly the odds of a good outcome (grade of 0–2) measured by mRS at 3 months [Fig. 3a, lower eGFR as reference, ordinal regression odds ratio, OR = 0.32 (confidence interval, CI, 95% 0.10–0.56), *p* = 0.005] and higher risk of hemorrhagic transformation [Fig. 3b, logistic regression OR 0.97 (CI95% 0.95–0.99), *p* = 0.026].

Figure 4 depicts outcome accordingly to CA status. Higher LF gain (worse CA) also increased the odds of a poor outcome (mRS > 2) [Fig. 4a, ipsilateral OR = 2.18 (CI95% 1.01–5.28), *p* = 0.048; fig. 4d, contralateral OR = 2.17 (CI95% 1.12–4.17), *p* = 0.022], and the risk of developing hemorrhagic transformation [Fig. 4b, ipsilateral OR = 1.98 (CI95% 1.002–8.12), Fig. 4e, p=0.042; contralateral OR = 2.79 (CI95% 1.03–7.56), *p* = 0.043].

An ordinal regression model with interaction between decreasing eGFR and increasing LF gain (Fig. 5a and d) found that both factors combine to progressively reducing the odds of a good functional outcome [ipsilateral OR = 4.39 (CI95% 3.15–25.6), *p* = 0.019; contralateral OR = 8.15 (CI95% 4.15–15.6), *p* = 0.002]. Multinomial logistic regression also found an interaction between lower eGFR and higher LF gain with an increased risk of hemorrhagic transformation that was only statistically significant on the contralateral side [Fig. 5b and e, ipsilateral OR = 3.48 (CI95% 0.60–24.0), *p* = 0.132; contralateral OR = 6.43 (CI95% 1.40–32.1), *p* = 0.034].

## Discussion

Our data shows significant association between impaired cerebral autoregulation (higher dynamic CA gain values) assessed during acute ischemic stroke (< 6 h) and impaired renal function (lower glomerular filtration rate) independent of age, vascular risk factors and relevant laboratory metabolic control parameters. Moreover, both impaired renal function and impaired cerebral autoregulation (i.e. high gain values) were associated with greater white matter abnormalities and with reduced likelihood of a good functional outcome (scale of 0–2) as assessed by mRS at 3 months.

To understand why impaired renal function might be linked to impaired cerebral autoregulation, we need to consider the physiology of autoregulation. Renal autoregulation adjusts vascular resistance to stabilize renal blood flow and filtration rate during changes in renal perfusion pressure [7]. Basically, as pressure changes stretch the smooth muscles of the afferent and cortical arterioles, a myogenic response occurs to adjust the vascular resistance to maintain flow. Since blood flow in a vascular bed is determined by perfusion pressure and resistance (Blood flow = perfusion pressure/vascular resistance), if perfusion pressure increases, stretching the smooth muscle surrounding the arterioles, the arterioles will then constrict to increase resistance and maintain the ratio of pressure to resistance and thus maintain blood flow [7]. In fact, renal autoregulation status has been well documented by the same method of TFA we used to assess dynamic CA. This method quantifies the transfer of blood pressure changes into flow change, thus high gain means that pressure changes are causing flow changes, and thus autoregulation is impaired. In the kidney, previous work in hypertensive rats with adenine-induced chronic renal failure has shown that gain is increased in the < 0.1 Hz band, suggesting higher gain is an indicator of impaired renal autoregulation [35].

The same characteristics are found in the cerebrovasculature with increased gain in the low-frequency range associated with impaired cerebral autoregulation [10, 20]. In fact, myogenic autoregulation is widely distributed over various vascular beds including the brain, coronary, pulmonary, mesenteric, and skeletal muscle [8].

Considering the similarities in the characteristics of autoregulation in the kidney and brain, one could propose that microvascular damage (e.g. caused by hypertension, diabetes) that causes impairment of kidney function [7, 16] could also affect the brain in a similar fashion. Support for this idea is found in stroke-prone spontaneously-hypertensive rats that have a genetic predisposition to rapid glomerulosclerosis and stroke [36]. In fact, in these rats, both renal autoregulation (assessed by TFA) and cerebral autoregulation are impaired prior to the development of stroke and death [37, 38]. Examination of the histopathology of cerebral arteries in the spontaneously hypertensive rats have also shown medial hypertrophy and remodeling, [39] that are hallmarks of small vessel disease, as in humans [17]. Thus it would appear that the stroke-prone spontaneously hypertensive rats show impairments of autoregulation in both the kidney and brain, prior to stroke. These data would suggest, in this animal model, that impairment of autoregulation is occurring in multiple vascular beds.

Taken together, this would suggest that if patients demonstrate less effective autoregulation in the cerebral vasculature (high gain) they would likely also have impaired renal myogenic autoregulation, which would cause glomerular dysfunction.

One interesting finding was that previous CCB use was correlated to increased LF gain values in both cerebral hemispheres as well as reduced eGFR. A possible explanation for this finding lies in the effect of CCB on autoregulation. In fact, previous work has demonstrated that CCB impairs the myogenic response in the kidney in animal models [7, 40] and perhaps in humans [41, 42]. CCB block the L-type voltage-gated calcium channels that are activated by the stretch of the arterial vessel wall [7]. Thus changes in pressure that result in changes in stretch of the walls will no longer activate the release of calcium and contraction of smooth muscle to modulate vascular resistance to maintain flow, which we would expect to result in an increased gain (i.e. worse autoregulation). The finding that CCB are associated with increased gain and poor eGFR may be due to CCB impairing autoregulation in both organs (brain and kidney), consistent with our hypothesis. However, the effect of CCB on cerebral autoregulation in humans is not as clear with studies, using measures other than TFA, showing impaired autoregulation, [41–43] while another study found no change in TFA derived gain in 8 healthy individuals [44].

Our finding that both low eGFR and high gain were significantly associated with severe WML grade does suggest that impaired autoregulation in the brain and kidney may be associated. A suggested mechanism for this is that microvascular damage is the basis of this autoregulatory dysfunction. Our work is the first to demonstrate that low eGFR is significantly associated with high gain and severe WML. From our data, we cannot rule out if WML cause cerebral autoregulation impairment or the opposite. Previous work has demonstrated that WML are correlated with cerebral autoregulation efficiency [14]. However, we hypothesize that both WML, as well as worse cerebral autoregulation, are expressions of a systemic microvascular dysfunction that can also impair renal function. Further prospective studies could untangle what’s cause or consequence.

Another finding in our study was the relationship between high gain, low eGFR and hemorrhagic transformation. Higher gain values reflect less effectiveness in the dampening capability of cerebral resistance vessels in the insonated vascular territory to ABP oscillations [6]. These dysfunctional vessels disrupt Starling’s principle by increasing the hydrostatic pressure across the capillary bed and contribute to the existing blood-brain barrier leakage [45]. Therefore, this might explain the association of higher gain and the risk of hemorrhagic transformation and cerebral edema [27]. Also, high gain values have been implicated in acute intracerebral hemorrhage [21, 46] and hematoma expansion [46].

In the infarcted hemisphere, gain had more non-significant relationships with outcome measurements. This can be observed by inspecting figs. 4 and 5. One obvious explanation is that some patients had occluded MCA at infarct side and CA cannot be assessed at infarct side in the severe cases. Therefore, gain apparently had weaker associations with outcome measures. A second possibility may be that the gain in the ischemic hemisphere is artificially lowered and cerebral autoregulation falsely normalized due to the increased vascular resistance caused by microthrombosis [47] and/or cytotoxic edema [48] in the infarcted area [49]. Clearly, much more work on the physiological determinants influencing each TFA parameter is needed.

One possible confounder on ABP variability and transfer function estimates of CA is the effect of atrial fibrillation. To address this we compared values between patients with and without atrial fibrillation and found no statistical difference between groups, suggesting atrial fibrillation did not have a significant effect on our estimates of ABP variability or CA.

The small sample is a limitation of this study. Also, we cannot rule out that this brain-kidney interaction is specific of a stroke population, rather than present in all individuals including healthy subjects since we don’t have a control group. Other angiographic imaging modalities (CT or MRI) are more accurate in excluding significant stenosis and since we relied mainly on ultrasound exams this may be a possible limitation. ABP was assessed using non-invasive oscillometric as well as finger photoplethysmography which are known to have limitations but generally provide a relatively accurate estimate of ABP [10].

Our results show that CA assessment may be an important factor to consider in advancing treatment of acute cerebrovascular disease. Given our finding that impaired CA is associated with worse functional outcome, further work is needed to verify and explore the clinical implications of our findings. Future studies could examine CA in ischemic stroke to determine its time course. In addition, longitudinal studies examining CA in a high-risk population could shed light on a new indicator of stroke risk. Studies assessing the effect of treatments targeting those with impaired CA could determine if the poor functional outcome could be mitigated by a treatment that targets cerebral perfusion for example. Finally, longitudinal studies would also be helpful in understanding if this CA impairment is acute or a chronic condition in these stroke patients.

## Conclusions

In conclusion, our findings provide support that early autoregulatory impairment of cerebral small vessels, as well as renal vessels, may be a possible mechanism linking low renal function with poor outcome of ischemic stroke. Rapid assessment of serum markers of renal dysfunction could be used as surrogates of cerebral microvascular function integrity which may help identify high-risk individuals for complications like hemorrhagic transformation and possibly provide a therapeutic target in the future.

## Additional file

Additional file 1: **Table S4.** Relationship between Cerebral Autoregulation Transfer Function parameters (Coherence, Gain and Phase) and demographic, clinical and laboratorial variables at acute stroke (within 6 h) explorer with linear regression analysis (corrected Beta and 95% interval of confidence intervals. **Table S5.** Relationship between Cerebral Autoregulation Transfer Function parameters (Coherence, Gain and Phase) and demographic, clinical and laboratorial variables at chronic stroke (3 months) explorer with linear regression analysis (corrected Beta and 95% interval of confidence intervals. (DOCX 60 kb)

## Abbreviations

ABP: Arterial blood pressure
CA: Cerebral autoregulation
CBFV: Cerebral blood flow velocity
CKD: Chronic kidney disease
CVRi: Cerebrovascular resistance
eGFR: Estimated glomerular filtration rate
HDL: High-density lipoprotein cholesterol
HgA1C: Glycated hemoglobin
LDL: Low-density lipoprotein cholesterol
MCA: Middle cerebral artery
mRS: modified Rankin Scale
NIHSS: National Institute of Health Stroke Scale
TFA: Transfer function analysis
