# Supplementary material for: Chronic kidney disease and poor outcomes in ischemic stroke: is impaired cerebral autoregulation the missing link?
Source: BMC Neurol. 2018 Mar 2;18:21. doi: 10.1186/s12883-018-1025-4 (PMC5834853; doi:10.1186/s12883-018-1025-4)
Supplement: Supplementary file 1 — Table S4. Relationship between Cerebral Autoregulation Transfer Function parameters (Coherence, Gain and Phase) and demographic, clinical and laboratorial variables at acute stroke (within 6 h) explorer with linear regression analysis (corrected Beta and 95% interval of confidence intervals. Table S5. Relationship between Cerebral Autoregulation Transfer Function parameters (Coherence, Gain and Phase) and demographic, clinical and laboratorial variables at chronic stroke (3 months) explorer with linear regression analysis (corrected Beta and 95% interval of confidence intervals. (DOCX 60 kb) [file 12883_2018_1025_MOESM1_ESM.docx]

**Additional file 1**

**Table S4** Relationship between Cerebral Autoregulation Transfer Function parameters (Coherence, Gain and Phase) and demographic, clinical and laboratorial variables at acute stroke (within 6 hours) explorer with linear regression analysis (corrected Beta and 95% interval of confidence intervals.

|  | Beta coefficients and estimated 95% confidence interval | | | | | |
| --- | --- | --- | --- | --- | --- | --- |
|  | Phase (degrees) | | Gain (%/mm Hg) | | Coherence (a.u.) | |
| *Ipsilateral* | LF band | HF band | LF band | HF band | LF band | HF band |
| Sex | – 0.15 (– 23.4, 13.4) | 0.21 (– 6.34, 17.3) | 0.08 (– 0.27, 0.38) | 0.02 (– 0.26, 0.87) | – 0.04 (– 0.11, 0.14) | – 0.02 (– 0.11, 0.14) |
| Age, years | 0.02 (– 0.61, 0.66) | 0.05 (– 0.38, 0.47) | 0.28 (– 0.01, 0.02) | 0.34 (– 0.01, 0.02) | – 0.08 (– 0.01, 0.04) | – 0.04 (– 0.01, 0.04) |
| BMI, kg/m^2^ | 0.15 (– 0.93, 2.17) | – 0.24 (– 1.46, 0.48) | 0.02 (– 0.02, 0.03) | 0.02 (– 0.04, 0.01) | 0.10 (– 0.01, 0.02) | 0.06 (– 0.01, 0.02) |
| Prev. Stroke | 0.20 (– 11.2, 37.8) | – 0.06 (– 19.1, 14.8) | – 0.16 (– 0.62, 0.29) | – 0.22 (– 0.70, 0.09) | 0.03 (– 0.19, 0.21) | 0.02 (– 0.19, 0.21) |
| Prev. MI | 0.05 (– 30.6, 39.7) | 0.29 (– 9.65, 44.2) | 0.49 (0.13, 1.45) | 0.15 (– 0.33, 1.09) | 0.21 (– 0.10, 0.42) | 0.17 (– 0.10, 0.42) |
| AF | 0.30 (– 3.7, 30.0) | – 0.35 (– 12.7, 11.0) | 0.15 (– 2.15, 0.42) | 0.26 (– 0.19, 0.53) | – 0.11 (– 0.20, 0.06) | – 0.11 (– 0.20, 0.06) |
| Hypertension | – 0.10 (– 28.5, 17.6) | – 0.04 (– 14.3, 11.3) | 0.14 (– 5.45, 13.6) | 0.10 (– 0.21, 0.42) | 0.01 (– 0.13, 0.14) | 0.10 (– 0.03, 0.11) |
| Diabetes | – 0.19 (– 30.3, 12.3) | – 0.03 (– 14.3, 4.43) | 0.32 (– 0.09, 0.56) | 0.09 (– 0.20, 0.38) | – 0.19 (–0.20, 0.06) | 0.07 (– 0.05, 0.10) |
| Dyslipidemia | – 0.04 (– 26.2, 22.3) | – 0.21 (– 20.3, 4.8) | – 0.07 (– 0.40, 0.31) | – 0.02 (– 0.35, 0.31) | 0.11(– 0.10, 0.20) | 0.23 (– 0.10, 0.34) |
| Tobacco | 0.09 (– 25.0, 37.7) | 0.19 (– 19.4, 6.96) | – 0.13 (– 0.59, 0.33) | – 0.16 (– 0.67, 0.19) | 0.14 (– 0.11, 0.25) | 0.14 (– 0.11, 0.25) |
| Antiplatelet | 0.08 (– 17.7, 25.5) | – 0.45 (–21.7, –0.83) | 0.04 (– 0.29, 0.35) | 0.00 (– 0.28, 0.28) | – 0.11 (– 0.17, 0.09) | – 0.12 (– 0.17, 0.09) |
| Statin | 0.19 (– 12.5, 30.6) | – 0.42 (– 21.5, 0.09) | 0.06 (– 0.29, 0.37) | 0.04 (– 0.27, 0.28) | – 0.14 (– 0.20, 0.07) | – 0.10 (– 0.21, 0.06) |
| Num. antiHT | – 0.24 (– 13.5, 4.1) | 0.33 (– 1.12, 8.03) | * 0.17 (0.05, 0.23) | 0.30 (– 0.03, 0.34) | 0.18 (– 0.06, 0.19) | 0.32 (– 0.02, 0.34) |
| Beta-Blocker | – 0.20 (– 1.5, 0.6) | 0.39 (– 4.22, 20.6) | 0.17 (– 0.29, 0.45) | 0.15 (– 0.15, 0.51) | 0.22 (0.06, 0.25) | 0.12 (0.10, 0.27) |
| ACEI/ARB | 0.12 (– 18.0, 30.2) | 0.06 (– 14.2, 10.9) | 0.19 (– 0.19, 0.56) | 0.11 (– 0.17, 0.39) | 0.13 (– 0.09, 0.19) | – 0.05 (– 0.08, 0.05) |
| CCB | – 0.32 (– 45.6, 7.5) | – 0.32 (– 45.6, 7.5) | *† 0.54 (0.21, 0.73) | * †0.40 (0.07, 1.09) | 0.17 (– 0.09, 0.26) | 0.17 (– 0.05, 0.12) |
| Large Vessel | – 0.13 (– 35.6, 20.0) | – 0.13 (– 23.6, 14.0) | – 0.28 (– 0.62, 0.18) | – 0.15 (– 0.66, 0.20) | – 0.10 (– .25, 0.14) | 0.05 (– 0.19, 0.25) |
| Cardioembolic | 0.30 (– 6.7, 35.1) | – 0.22 (– 12.3, 34.1) | 0.15 (– 0.21, 0.41) | 0.26 (– 0.02, 0.53) | – 0.18 (– 0.20, 0.06) | – 0.25 (– 0.23, 0.14) |
| Small vessel | 0.13 (– 27.0, 47.6) | 0.32 (– 14.3, 5.46) | 0.02 (– 0.53, 0.58) | – 0.27 (– 0.81, 0.02) | 0.10 (– 0.12, 0.21) | 0.01 (– 0.02, 0.03) |
| NIHSS | – 0.07 (– 2.33, 1.72) | 0.14 (– 0.77, 1.40) | * 0.03 (0.002, 0.04) | * 0.04 (0.01, 0.07) | 0.20 (– 0.01, 0.02) | – 0.03 (– 0.05, 0.01) |
| Leukoariosis | – 0.12 (– 28.0, 16.7) | 0.20 (– 2.67, 6.89) | 0.24 (– 0.01, 0.24) | 0.28 (– 0.13, 0.22) | – 0.19 (– 0.08, 0.02) | – 0.19 (– 0.08, 0.02) |
| Creatinine | – 0.07 (– 9.83, 7.46) | 0.30 (– 1.58, 7.34) | * 0.17 (0.05, 0.26) | * 0.23 (0.07, 0.35) | 0.05 (– 0.02, 0.10) | 0.15 (– 0.02, 0.23) |
| eGFR | – 0.05 (– 0.31, 3.24) | – 0.21 (– 0.22, 0.08) | *†–0.07 (–0.08,–0.01) | *†–0.02 (–0.02, –0.01) | – 0.03 (0.01, –0.01) | – 0.03 (0.01, 0.00) |
| CRP | 0.01 (– 1.31, 1.42) | – 0.24 (– 1.10, 0.33) | 0.24 (– 0.01, 0.03) | 0.05 (– 0.01, 0.01) | 0.08 (– 0.01, 0.01) | 0.04 (– 0.02, 0.05) |
| Cholesterol | 0.07 (– 0.20, 0.31) | – 0.63 (– 0.73, 0.51) | – 0.37 (– 0.05, 0.00) | – 0.33 (– 0.02, 0.02) | 0.27 (– 0.01, 0.01) | 0.11 (– 0.04, 0.21) |
| LDL | 0.14 (– 0.17, 0.32) | 0.27 (– 0.66, 0.44) | – 0.35 (– 0.03, 0.01) | – 0.23 (– 0.01, 0.02) | – 0.27 (– 0.01, 0.01) | – 0.23 (– 0.01, 0.01) |
| HDL | – 0.17 (– 0.01, 0.3) | 0.23 (– 0.26, 0.35) | – 0.18 (– 0.02, 0.01) | – 0.26 (– 0.04, 0.01) | – 0.14 (– 0.01, 0.03) | – 0.25 (– 0.01, 0.03) |
| TG | 0.28 (– 1.21, 0.60) | 0.36 (– 1.34, 1.60) | – 0.07 (– 0.03, 0.02) | 0.13 (– 0.01, 0.10) | – 0.21 (– 0.03, 0.02) | – 0.22 (– 0.03, 0.02) |
| Glucose | 0.13 (– 0.07, 0.15) | – 0.17 (– 0.08, 0.04) | 0.04 (– 0.01, 0.02) | 0.05 (– 0.02, 0.02) | – 0.42 (–0.02, 0.01) | – 0.10 (–0.06, 0.04) |
| HbA1C | 0.02 (– 6.73, 6.90) | – 0.14 (– 5.13, 3.15) | – 0.19 (– 1.29, 0.06) | – 0.20 (– 0.18, 0.04) | – 0.07 (– 0.08, 0.00) | – 0.05 (– 0.06, 0.00) |
| Mean BP | 0.28 (– 1.21, 0.60) | 0.28 (– 1.21, 0.60) | * –0.34 (– 0.02,0.00) | *†–0.02(– 0.02, –0.01) | – 0.01 (– 0.01, 0.01) | 0.06 (– 0.27, 0.37) |
| Heart rate | 0.13 (– 0.33, 1.26) | – 0.10 (– 1.23, 0.23) | – 0.001 (– 0.03, 0.03) | – 0.15 (– 0.02, 0.01) | – 0.10 (– 0.01, 0.00) | – 0.09 (– 0.10, 0.01) |
| EtCO2 | 0.31 (– 0.10, .34) | 0.13 (– 0.13, 2.31) | – 0.04 (– 0.08, 0.05) | – 0.29 (– 0.04, 0.00) | – 0.13 (– 0.01, 0.01) | – 0.01 (– 0.01, 0.01) |
| *Contralateral* |  |  |  |  |  |  |
| Sex | 0.07 (– 13.6, 21.1) | 0.24 (– 2.14, 14.0) | 0.02 (– 0.26, 0.87) | – 0.06 (– 1.21, 1.42) | 0.02 (– 0.26, 0.31) | – 0.02 (– 0.11, 0.14) |
| Age, years | – 0.23 (– 1.16, 0.21) | 0.17 (– 0.17, 0.50) | * 0.02 (0.01, 0.02) | * 0.06 (0.03, 0.08) | 0.01 (– 0.01, 0.02) | 0.02 (– 0.01, 0.04) |
| BMI, kg/m^2^ | – 0.13 (– 2.50, 1.12) | – 0.05 (– 1.00, 0.75) | 0.02 (– 0.04, 0.01) | – 0.05 (– 0.06, 0.01) | – 0.01 (– 0.05, 0.02) | 0.01 (– 0.01, 0.02) |
| Prev. Stroke | 0.01 (– 22.8, 24.2) | – 0.04 (– 12.4, 10.1) | – 0.40 (– 0.70, 0.09) | 0.20 (– 0.53, 1.10) | – 0.22 (– 0.70, 0.09) | 0.02 (– 0.19, 0.21) |
| Prev. MI | – 0.08 (– 66.3, 40.3) | 0.23 (– 2.47, 25.3) | 0.15 (– 0.33, 1.09) | 0.19 (–0.25, 1.47) | 0.15 (– 0.33, 1.09) | 0.17 (– 0.10, 0.42) |
| AF | 0.15 (– 9.61, 24.0) | 0.28 (– 1.27, 14.8) | 0.26 (– 0.19, 0.53) | * 0.46 (0.26, 0.83) | 0.26 (– 0.02, 0.53) | – 0.11 (– 0.20, 0.06) |
| Hypertension | – 0.29 (– 32.6, 6.85) | 0.14 (– 5.45, 13.6) | 0.10 (– 0.21, 0.42) | 0.06 (– 0.31, 0.447) | 0.09 (– 0.22, 0.42) | 0.10 (– 0.03, 0.11) |
| Diabetes | – 0.31 (– 37.4, 1.9) | 0.12 (– 6.08, 4.93) | 0.09 (– 0.20, 0.38) | – 0.01(–0.33, 0.36) | 0.09 (– 0.20, 0.38) | 0.19 (0.05, 0.10) |
| Dyslipidemia | – 0.34 (– 30.2, 8.35) | – 0.12 (– 5.31, 2.43) | – 0.02 (– 0.35, 0.31) | – 0.19 (–0.62, 0.13) | – 0.02 (– 0.35, 0.31) | 0.23 (– 0.10, 0.34) |
| Tobacco | – 0.12 (– 37.0, 17.5) | 0.23 (– 24.4, 2.24) | – 0.16 (– 0.67, 0.19) | – 0.20 (– 0.85, 0.15) | – 0.16 (– 0.67, 0.19) | 0.14 (– 0.11, 0.25) |
| Antiplatelet | 0.09 (– 26.4, 22.1) | – 0.45 (–21.7, –0.83) | 0.00 (– 0.28, 0.28) | 0.08 (– 0.24, 0.43) | 0.00 (– 2.83, 2.83) | – 0.12 (– 0.17, 0.09) |
| Statin | 0.14 (– 10.0, 25.4) | 0.18 (– 4.35, 12.3) | 0.04 (– 0.27, 0.28) | 0.15 (– 0.12, 0.33) | 0.01 (– 0.27, 0.30) | – 0.10 (– 0.21, 0.06) |
| Num. antiHT | – 0.16 (– 10.5, 3.57) | 0.33 (– 1.12, 8.03) | * 0.38 (0.05, 0.27) | 0.43 (–0.19, 0.26) | 0.23 (– 0.05, 0.05) | 0.32 (– 0.02, 0.34) |
| Beta-Blocker | – 0.07 (– 23.5, 15.5) | 0.45 (– 4.34, 14.4) | 0.15 (– 0.15, 0.51) | 0.27 (–0.02, 0.75) | 0.16 (– 0.15, 0.51) | 0.12 (0.10, 0.27) |
| ACEI/ARB | 0.11 (– 12.0, 23.2) | 0.16 (– 20.1, 3.4) | 0.11 (– 0.17, 0.39) | – 0.04 (– 0.38, 0.29) | 0.11 (– 0.17, 0.39) | – 0.05 (– 0.08, 0.05) |
| CCB | – 0.15 (– 34.5, 10.2) | – 0.32 (– 45.6, 7.5) | *† 0.40 (0.16, 0.81) | 0.25 (– 0.04, 0.76) | 0.14 (– 0.06, 0.24) | 0.17 (– 0.05, 0.12) |
| Large Vessel | 0.14 (– 13.3, 33.1) | 0.10 (– 14.6, 4.0) | – 0.15 (– 0.66, 0.20) | – 0.27 (– 0.95, 0.03) | – 0.15 (– 0.65, 0.20) | 0.05 (– 0.19, 0.25) |
| Cardioembolic | 0.15 (– 9.64, 24.5) | 0.32 (– 23.3, 3.31) | 0.26 (– 0.02, 0.53) | 0.45 (0.22, 0.87) | – 0.11 (– 0.18, 0.02) | – 0.25 (– 0.23, 0.14) |
| Small vessel | 0.19 (– 58.8, 16.4) | 0.23 (– 3.3, 15.3) | – 0.27 (– 0.81, 0.02) | – 0.27 (– 0.96, 0.02) | 0.29 (– 0.12, 0.26) | 0.01 (– 0.02, 0.03) |
| NIHSS | – 0.25 (– 2.31, 0.34) | 0.14 (– 0.77, 1.40) | * 0.37 (0.01, 0.04) | * 0.41 (0.01, 0.54) | 0.08 (– 0.01, 0.01) | – 0.03 (– 0.05, 0.01) |
| Leukoariosis | – 0.08 (– 8.47, 5.39) | 0.20 (– 2.67, 6.89) | * 0.28 (0.00, 0.22) | 0.20 (– 0.04, 0.22) | – 0.19 (– 0.08, 0.02) | – 0.19 (– 0.08, 0.02) |
| Creatinine | – 0.05 (– 9.8, 7.1) | 0.20 (– 2.21, 10.2) | * 0.39 (0.07, 0.35) | * 0.41 (0.09, 0.41) | 0.16 (– 0.02, 0.23) | 0.15 (– 0.02, 0.14) |
| eGFR | – 0.04 (– 0.19, 0.25) | – 0.29 (– 0.12, 0.09) | *†–0.44 (–0.01, 0.00) | *†–0.51 (–0.01, 0.00) | – 0.03 (0.01, –0.01) | – 0.02 (0.01, 0.00) |
| CRP | 0.14 (– 0.35, 0.82) | 0.04 (– 0.25, 0.32) | 0.05 (– 0.01, 0.01) | 0.02 (– 0.12, 0.04) | 0.08 (– 0.01, 0.01) | 0.04 (– 0.02, 0.05) |
| Cholesterol | – 0.25 (– 0.29, 0.04) | – 0.05 (– 0.52, 0.54) | – 0.33 (– 0.02, 0.02) | – 0.46 (– 0.11, 0.12) | 0.21 (– 0.01, 0.01) | 0.11 (– 0.04, 0.21) |
| LDL | – 0.28 (– 0.34, 0.03) | – 0.35 (– 1.08, 0.40) | – 0.23 (– 0.01, 0.02) | – 0.25 (– 0.02, 0.01) | – 0.24 (– 0.01, 0.01) | – 0.23 (– 0.01, 0.01) |
| HDL | – 0.07 (– 0.97, 0.65) | 0.14 (– 0.34, 0.12) | – 0.26 (– 0.04, 0.01) | – 0.15 (– 0.03, 0.04) | – 0.14 (– 0.04, 0.02) | – 0.25 (– 0.01, 0.03) |
| TG | – 0.22 (– 1.96, 0.04) | 0.34 (– 1.23, 1.02) | 0.13 (– 0.01, 0.10) | 0.02 (– 0.12, 0.24) | – 0.21 (– 0.03, 0.02) | – 0.22 (– 0.03, 0.02) |
| Glucose | 0.01 (– 0.05, 0.13) | – 0.06 (– 0.05, 0.05) | 0.05 (– 0.02, 0.02) | 0.23 (– 0.12, 0.02) | – 0.42 (–0.02, 0.01) | – 0.10 (–0.06, 0.04) |
| HbA1C | 0.14 (– 4.1, 10.5) | – 0.22 (– 4.34, 1.15) | – 0.20 (– 0.18, 0.04) | 0.12 (– 0.10, 0.12) | 0.02 (– 0.10, 0.12) | – 0.05 (– 0.06, 0.00) |
| Mean BP | 0.17 (– 0.26, 0.84) | 0.21 (– 1.23, 1.01) | *†–0.50 (–0.02, –0.01) | *†–0.47 (–0.03, –0.01) | – 0.04 (–0.04, 0.01) | – 0.05 (– 0.07, 0.02) |
| Heart rate | – 0.04 (– 0.58, 1.03) | – 0.10 (– 2.33, 0.12) | – 0.15 (– 0.02, 0.01) | – 0.32 (– 0.01, 0.13) | – 0.12 (– 0.04, 0.01) | – 0.09 (– 0.10, 0.01) |
| EtCO2 | 0.13 ( 0.14, 2.56) | – 0.25 (– 0.23, 3.12) | – 0.29 (– 0.04, 0.00) | – 0.24 (– 0.01, 0.12) | – 0.12 (– 0.02, 0.01) | – 0.11 (– 0.01, 0.01) |

Body-Mass Index (BMI), Transitory Ischemic Attack (TIA), Atrial fibrillation (AF), Congestive Heart Failure (CHF), Heart Disease (HD), Left ventricule (LV) hypertrophy (LVH) and ejection fraction (LVEF), angiotensin-conversion-enzyme inhibitor (ACEI), angiotensin receptor blocker (ARB), calcium channel blocker (CCB), number of anti-hypertensive drugs (Num. antiHT), middle cerebral artery (MCA), National Institutes of Health Stroke Scale (NIHSS); Large Vessel, Cardioembolic and small vessel are the etiologies of stroke; Leukoariosis refers to the grade of leukoencephalopathy calculated by van Swieten scale[^34^](#_ENREF_34) on 24-hours CT; Glomerular filtration rate (GFR) estimated by Cockcroft-Gault formula, Brain Natriuretic Peptide (BNP), C-reactive protein (CRP), Low-density lipoprotein (LDL), High-density lipoprotein (LDL), triglycerides (TG), glycated hemoglobin (HbA1C), arterial blood pressure (BP), cerebrovascular resistance index (CVRi), end-tidal carbon dioxide (EtCO2), low (LF, 0 – 0.15 Hz) and high (HF, 0.15 – 0.5 Hz) frequency spectral bands. For units see table 1.

* P< 0.01 for linear regression analysis, †P< 0.05 after adjustment to age, gender, NIHSS and all independent variables significantly associated with the TFA parameter in univariate analysis.

**Table S5** Relationship between Cerebral Autoregulation Transfer Function parameters (Coherence, Gain and Phase) and demographic, clinical and laboratorial variables at chronic stroke (3 months) explorer with linear regression analysis (corrected Beta and 95% interval of confidence intervals.

|  | Beta coefficients and estimated 95% confidence interval | | | | | |
| --- | --- | --- | --- | --- | --- | --- |
|  | Phase (degress) | | Gain (%/mm Hg) | | Coherence (a.u.) | |
| *Ipsilateral* | LF band | HF band | LF band | HF band | LF band | HF band |
| Sex | – 0.24 (– 39.4, 23.4) | 0.21 (– 6.34, 17.3) | 0.17 (– 0.12, 0.34) | – 0.06 (– 0.32, 0.22) | – 0.04 (– 0.11, 0.14) | – 0.02 (– 0.11, 0.14) |
| Age, years | – 0.17 (–1.04, 0.36) | 0.05 (– 0.38, 0.47) | 0.06 (0.00, 0.01) | 0.25 (– 0.02, 0.03) | – 0.08 (– 0.01, 0.04) | – 0.04 (– 0.01, 0.04) |
| BMI, kg/m^2^ | 0.00 (– 1.62, 1.607) | – 0.24 (– 1.46, 0.48) | 0.02 (– 0.02, 0.03) | 0.01 (– 0.01, 0.03) | 0.10 (– 0.01, 0.02) | 0.06 (– 0.01, 0.02) |
| Prev. Stroke | 0.02 (– 31.2, 19.8) | – 0.06 (– 19.1, 14.8) | – 0.08 (– 0.56, 0.12) | 0.02 (– 0.24, 0.23) | 0.03 (– 0.19, 0.21) | 0.02 (– 0.19, 0.21) |
| Prev. MI | 0.06 (– 40.6, 38.7) | 0.29 (– 9.65, 44.2) | – 0.10 (–1.05, 0.56) | 0.23 (0.23, 1.23) | 0.21 (– 0.10, 0.42) | 0.17 (– 0.10, 0.42) |
| AF | – 0.13 (– 23.7, 10.0) | – 0.35 (– 12.7, 11.0) | 0.05 (– 1.23, 0.46) | 0.34 (– 0.02, 0.54) | – 0.11 (– 0.20, 0.06) | – 0.11 (– 0.20, 0.06) |
| Hypertension | – 0.21 (– 28.5, 17.6) | – 0.04 (– 14.3, 11.3) | – 0.14 (– 5.12, 11.6) | – 0.02 (– 0.32, 0.27) | 0.01 (– 0.13, 0.14) | 0.10 (– 0.03, 0.11) |
| Diabetes | – 0.19 (– 30.3, 12.3) | – 0.03 (– 14.3, 4.43) | 0.21 (– 0.12, 0.26) | – 0.08 (– 0.35, 0.21) | – 0.19 (–0.20, 0.06) | 0.19 ( 0.05, 0.10) |
| Dyslipidemia | – 0.13 (– 33.2, 14.3) | – 0.21 (– 20.3, 4.8) | \0.12 (– 0.12, 0.34) | – 0.05 (–0.36, –0.27) | 0.11(– 0.10, 0.20) | 0.23 (– 0.10, 0.34) |
| Tobacco | 0.09 (– 25.0, 37.7) | 0.19 (– 19.4, 6.96) | 0.23 (– 0.51, 0.64) | 0.06 (– 0.32, 0.47) | 0.14 (– 0.11, 0.25) | 0.14 (– 0.11, 0.25) |
| Antiplatelet | 0.08 (– 17.7, 25.5) | – 0.45 (–21.7, –0.83) | 0.01 (– 0.10, 0.12) | 0.01 (– 0.34, 0.34) | – 0.11 (– 0.17, 0.09) | – 0.12 (– 0.17, 0.09) |
| Statin | 0.19 (– 12.5, 30.6) | – 0.42 (– 21.5, 0.09) | 0.02 (– 0.02, 0.37) | 0.03 (– 0.23, 0.24) | – 0.14 (– 0.20, 0.07) | – 0.10 (– 0.21, 0.06) |
| Num. antiHT | – 0.24 (– 13.5, 4.1) | 0.33 (– 1.12, 8.03) | 0.53 (0.04, 0.26) | 0.22 (–0.04, 0.21) | 0.18 (– 0.06, 0.19) | 0.32 (– 0.02, 0.34) |
| Beta-Blocker | – 0.20 (– 1.5, 0.6) | 0.39 (– 4.22, 20.6) | 0.17 (– 0.29, 0.45) | 0.22 (– 0.12, 0.64) | 0.22 (0.06, 0.25) | 0.12 (0.10, 0.27) |
| ACEI/ARB | 0.12 (– 18.0, 30.2) | 0.06 (– 14.2, 10.9) | 0.19 (– 0.19, 0.56) | 0.017 (– 0.24, 0.57) | 0.13 (– 0.09, 0.19) | – 0.05 (– 0.08, 0.05) |
| CCB | – 0.32 (– 45.6, 7.5) | – 0.32 (– 45.6, 7.5) | 0.23 (– 0.05, 0.46) | 0.25 (0.04, 0.23) | 0.17 (– 0.09, 0.26) | 0.17 (– 0.05, 0.12) |
| Large Vessel | – 0.13 (– 35.6, 20.0) | – 0.13 (– 23.6, 14.0) | – 0.28 (– 0.62, 0.18) | – 0.19 (– 0.68, 0.24) | – 0.10 (– .25, 0.14) | 0.05 (– 0.19, 0.25) |
| Cardioembolic | 0.30 (– 6.7, 35.1) | – 0.22 (– 12.3, 34.1) | 0.15 (– 0.21, 0.41) | 0.28 (– 0.19, 0.90) | – 0.18 (– 0.20, 0.06) | – 0.25 (– 0.23, 0.14) |
| Small vessel | 0.13 (– 27.0, 47.6) | 0.32 (– 14.3, 5.46) | 0.02 (– 0.53, 0.58) | 0.23 (– 1.43, 0.47) | 0.10 (– 0.12, 0.21) | 0.01 (– 0.02, 0.03) |
| NIHSS | – 0.07 (– 2.33, 1.72) | 0.14 (– 0.77, 1.40) | 0.34 (– 0.01, 0.05) | 0.17 (– 0.01, 0.03) | 0.20 (– 0.01, 0.02) | – 0.03 (– 0.05, 0.01) |
| Leukoariosis | – 0.12 (– 28.0, 16.7) | 0.20 (– 2.67, 6.89) | 0.24 (– 0.01, 0.24) | – 0.07 (– 0.56, 0.23) | – 0.19 (– 0.08, 0.02) | – 0.19 (– 0.08, 0.02) |
| Creatinine | – 0.07 (– 9.83, 7.46) | 0.30 (– 1.58, 7.34) | 0.04 (0.01, 0.40) | 0.23 (0.05, 0.23) | 0.24 (– 0.02, 0.10) | 0.15 (– 0.02, 0.23) |
| eGFR | – 0.05 (– 0.31, 3.24) | – 0.21 (– 0.22, 0.08) | – 0.05 (–0.36, –0.25) | – 0.23 (– 0.56, –0.34) | – 0.03 (0.01, –0.01) | – 0.03 (0.01, 0.00) |
| CRP | 0.01 (– 1.31, 1.42) | – 0.24 (– 1.10, 0.33) | 0.24 (– 0.01, 0.03) | 0.18 (– 0.03, 0.05) | 0.08 (– 0.01, 0.01) | 0.04 (– 0.02, 0.05) |
| Cholesterol | 0.07 (– 0.20, 0.31) | – 0.63 (– 0.73, 0.51) | – 0.37 (– 0.05, 0.00) | – 0.23 (– 0.12, 0.01) | 0.27 (– 0.01, 0.01) | 0.11 (– 0.04, 0.21) |
| LDL | 0.14 (– 0.17, 0.32) | 0.27 (– 0.66, 0.44) | – 0.35 (– 0.03, 0.01) | – 0.12 (– 0.12, 0.01) | – 0.27 (– 0.01, 0.01) | – 0.23 (– 0.01, 0.01) |
| HDL | – 0.17 (– 0.01, 0.3) | 0.23 (– 0.26, 0.35) | – 0.18 (– 0.02, 0.01) | 0.12 (– 0.01, 0.12) | – 0.14 (– 0.01, 0.03) | – 0.25 (– 0.01, 0.03) |
| TG | 0.28 (– 1.21, 0.60) | 0.36 (– 1.34, 1.60) | – 0.07 (– 0.03, 0.01) | – 0.04 (– 0.12, 0.12) | – 0.21 (– 0.03, 0.02) | – 0.22 (– 0.03, 0.02) |
| Glucose | 0.16 (– 0.07, 0.15) | – 0.17 (– 0.08, 0.04) | 0.04 (– 0.01, 0.02) | 0.09 (– 0.09, 0.12) | – 0.42 (–0.02, 0.01) | – 0.10 (–0.06, 0.04) |
| HbA1C | 0.02 (– 6.73, 6.90) | – 0.14 (– 5.13, 3.15) | – 0.29 (– 2.19, 0.12) | 0.12 (– 0.10, 0.12) | – 0.07 (– 0.08, 0.00) | – 0.05 (– 0.06, 0.00) |
| Mean BP | 0.28 (– 1.21, 0.60) | 0.28 (– 1.21, 0.60) | – 0.04 (– 0.01, 0.01) | *†–0.42 (–0.02, 0.01) | – 0.01 (– 0.01, 0.01) | 0.06 (– 0.27, 0.37) |
| Heart rate | 0.11 (– 0.79, 1.26) | 0.24 (– 1.23, 0.23) | – 0.20 (– 0.02, 0.03) | – 0.32 (– 0.01, 0.02) | – 0.10 (– 0.01, 0.00) | – 0.09 (– 0.10, 0.01) |
| EtCO2 | 0.20 (– 0.90, 2.34) | 0.13 (– 0.13, 2.31) | 0.24 (– 0.02, 0.10) | – 0.23 (– 0.10, 0.02) | – 0.13 (– 0.01, 0.01) | – 0.11 (– 0.01, 0.01) |
| *Contralateral* |  |  |  |  |  |  |
| Sex | 0.07 (– 13.6, 21.1) | 0.24 (– 2.14, 14.0) | 0.15 (– 0.14, 0.36) | – 0.06 (– 1.21, 1.42) | – 0.04 (– 0.11, 0.14) | – 0.02 (– 0.11, 0.14) |
| Age, years | 0.33 (– 1.04, 3.21) | 0.17 (– 0.17, 0.50) | 0.14 (– 0.01, 0.02) | 0.24 (0.02, 0.01) | – 0.08 (– 0.01, 0.04) | – 0.04 (– 0.01, 0.04) |
| BMI, kg/m^2^ | 0.06 (– 2.50, 2.60) | – 0.05 (– 1.00, 0.75) | – 0.11 (– 0.03, 0.02) | – 0.19 (– 0.06, 0.01) | 0.10 (– 0.01, 0.02) | 0.06 (– 0.01, 0.02) |
| Prev. Stroke | 0.01 (– 24.8, 34.2) | – 0.04 (– 12.4, 10.1) | – 0.15 (– 0.51, 0.19) | 0.13 (– 0.53, 1.10) | 0.03 (– 0.19, 0.21) | 0.02 (– 0.19, 0.21) |
| Prev. MI | – 0.10 (– 67.3, 50.1) | 0.23 (– 2.47, 25.3) | 0.15 (– 0.33, 1.09) | 0.19 (–0.25, 1.47) | 0.21 (– 0.10, 0.42) | 0.17 (– 0.10, 0.42) |
| AF | 0.05 (– 6.45, 15.0) | 0.28 (– 1.27, 14.8) | 0.31 (0.00, 0.48) | 0.39 (0.08, 0.63) | – 0.11 (– 0.20, 0.06) | – 0.11 (– 0.20, 0.06) |
| Hypertension | – 0.29 (– 32.6, 6.85) | 0.14 (– 5.45, 13.6) | – 0.03 (– 0.29, 0.25) | 0.06 (– 0.31, 0.447) | 0.01 (– 0.13, 0.14) | 0.10 (– 0.03, 0.11) |
| Diabetes | – 0.31 (– 37.4, 1.9) | 0.12 (– 6.08, 4.93) | – 0.03 (– 0.29, 0.23) | – 0.01(–0.33, 0.36) | – 0.19 (–0.20, 0.06) | 0.19 (– 0.05, 0.10) |
| Dyslipidemia | – 0.34 (– 30.2, 8.35) | – 0.12 (– 5.31, 2.43) | 0.05 (– 0.24, 0.34) | – 0.19 (–0.62, 0.13) | 0.11(– 0.10, 0.20) | 0.23 (– 0.10, 0.34) |
| Tobacco | – 0.12 (– 37.0, 17.5) | 0.23 (– 24.4, 2.24) | – 0.10 (– 0.47, 0.24) | – 0.20 (– 0.85, 0.15) | 0.14 (– 0.11, 0.25) | 0.14 (– 0.11, 0.25) |
| Antiplatelet | 0.09 (– 26.4, 22.1) | – 0.45 (–21.7, –0.83) | 0.00 (– 0.28, 0.28) | 0.08 (– 0.24, 0.43) | – 0.11 (– 0.17, 0.09) | – 0.12 (– 0.17, 0.09) |
| Statin | 0.14 (– 10.0, 25.4) | 0.18 (– 4.35, 12.3) | 0.01 (– 0.01, 0.03) | 0.15 (– 0.12, 0.33) | – 0.14 (– 0.20, 0.07) | – 0.10 (– 0.21, 0.06) |
| Num. antiHT | – 0.16 (– 10.5, 3.57) | 0.33 (– 1.12, 8.03) | 0.07 (– 0.08, 0.15) | 0.43 (–0.19, 0.26) | 0.18 (– 0.06, 0.19) | 0.32 (– 0.02, 0.34) |
| Beta-Blocker | 0.15 (–15.5, 23.1) | 0.45 (– 4.34, 14.4) | – 0.15 (– 0.53, 0.17) | 0.27 (–0.02, 0.75) | 0.22 (0.06, 0.25) | 0.12 (0.10, 0.27) |
| ACEI/ARB | 0.11 (– 12.0, 23.2) | 0.16 (– 20.1, 3.4) | 0.18 (– 0.17, 0.35) | – 0.04 (– 0.38, 0.29) | 0.13 (– 0.09, 0.19) | – 0.05 (– 0.08, 0.05) |
| CCB | – 0.15 (– 34.5, 10.2) | – 0.32 (– 45.6, 7.5) | 0.24 (0.15, 0.46) | 0.25 (– 0.02, 0.36) | 0.17 (– 0.09, 0.26) | 0.17 (– 0.05, 0.12) |
| Large Vessel | 0.14 (– 13.3, 33.1) | 0.10 (– 14.6, 4.0) | – 0.15 (– 0.66, 0.20) | – 0.23 (– 0.34, 0.12) | – 0.10 (– .25, 0.14) | 0.05 (– 0.19, 0.25) |
| Cardioemb. | 0.15 (– 9.64, 24.5) | 0.32 (– 23.3, 3.31) | 0.26 (– 0.01, 0.45) | 0.45 (0.22, 0.87) | – 0.18 (– 0.20, 0.06) | – 0.25 (– 0.23, 0.14) |
| Small vessel | 0.19 (– 58.8, 16.4) | 0.23 (– 3.3, 15.3) | – 0.15 (– 0.31, 0.23) | 0.24 (– 0.34, 0.02) | 0.10 (– 0.12, 0.21) | 0.01 (– 0.02, 0.03) |
| NIHSS | – 0.25 (– 2.31, 0.34) | 0.14 (– 0.77, 1.40) | 0.21 (– 0.01, 0.03) | 0.12 (0.01, 0.34) | 0.20 (– 0.01, 0.02) | – 0.03 (– 0.05, 0.01) |
| Leukoariosis | – 0.08 (– 8.47, 5.39) | 0.20 (– 2.67, 6.89) | 0.28 (0.00, 0.22) | 0.20 (– 0.04, 0.22) | – 0.19 (– 0.08, 0.02) | – 0.19 (– 0.08, 0.02) |
| Creatinine | – 0.05 (– 9.8, 7.1) | 0.20 (– 2.21, 10.2) | – 0.02 (–0.32, 0.33) | 0.02 (– 0.34, 0.34) | 0.24 (– 0.02, 0.10) | 0.15 (– 0.02, 0.23) |
| eGFR | – 0.04 (– 0.19, 0.25) | – 0.29 (– 0.12, 0.09) | 0.04 (– 0.03, 0.00) | – 0.12 (–0.01, 0.00) | – 0.03 (0.01, –0.01) | – 0.03 (0.01, 0.00) |
| CRP | 0.14 (– 1.35, 3.82) | 0.04 (– 0.25, 0.32) | 0.02 (– 0.01, 0.02) | 0.02 (– 0.12, 0.04) | 0.08 (– 0.01, 0.01) | 0.04 (– 0.02, 0.05) |
| Cholesterol | – 0.25 (– 0.29, 0.04) | – 0.05 (– 0.52, 0.54) | – 0.12 (– 0.04, 0.02) | – 0.46 (– 0.11, 0.12) | 0.27 (– 0.01, 0.01) | 0.11 (– 0.04, 0.21) |
| LDL | – 0.28 (– 0.34, 0.03) | – 0.35 (– 1.08, 0.40) | – 0.23 (– 0.05, 0.01) | – 0.25 (– 0.02, 0.01) | – 0.27 (– 0.01, 0.01) | – 0.23 (– 0.01, 0.01) |
| HDL | – 0.07 (– 0.97, 0.65) | 0.14 (– 0.34, 0.12) | – 0.12 (– 0.01, 0.03) | – 0.15 (– 0.03, 0.04) | – 0.14 (– 0.01, 0.03) | – 0.25 (– 0.01, 0.03) |
| TG | – 0.22 (– 1.96, 0.04) | 0.34 (– 1.23, 1.02) | 0.13 (– 0.01, 0.10) | 0.02 (– 0.12, 0.24) | – 0.21 (– 0.03, 0.02) | – 0.22 (– 0.03, 0.02) |
| Glucose | – 0.01 (– 0.11, 0.11) | – 0.06 (– 0.05, 0.05) | 0.05 (– 0.02, 0.02) | 0.23 (– 0.12, 0.02) | – 0.42 (–0.02, 0.01) | – 0.10 (–0.06, 0.04) |
| HbA1C | 0.06 (– 5.6, 7.7) | – 0.22 (– 4.34, 1.15) | – 0.20 (– 0.18, 0.04) | 0.12 (– 0.10, 0.12) | – 0.07 (– 0.08, 0.00) | – 0.05 (– 0.06, 0.00) |
| Mean BP | 0.17 (– 0.26, 0.84) | 0.21 (– 1.23, 1.01) | – 0.18 (–0.02, 0.01) | – 0.31 (–0.02, 0.00) | – 0.01 (– 0.01, 0.01) | 0.06 (– 0.27, 0.37) |
| Heart rate | 0.10 (– 0.58, 1.03) | – 0.10 (– 2.33, 0.12) | – 0.12 (– 0.01, 0.00) | – 0.23 (– 0.01, 0.12) | – 0.10 (– 0.01, 0.00) | – 0.09 (– 0.10, 0.01) |
| EtCO2 | 0.21 ( 0.00, 2.30) | – 0.25 (– 0.23, 3.12) | – 0.23 (– 0.05, 0.01) | – 0.23 (– 0.01, 0.17) | – 0.13 (– 0.01, 0.01) | – 0.11 (– 0.01, 0.01) |

Body-Mass Index (BMI), Transitory Ischemic Attack (TIA), Atrial fibrillation (AF), Congestive Heart Failure (CHF), Heart Disease (HD), Left ventricule (LV) hypertrophy (LVH) and ejection fraction (LVEF), angiotensin-conversion-enzyme inhibitor (ACEI), angiotensin receptor blocker (ARB), calcium channel blocker (CCB), number of anti-hypertensive drugs (Num. antiHT), middle cerebral artery (MCA), National Institutes of Health Stroke Scale (NIHSS); Large Vessel, cardioembolism (Cardioemb.) and small vessel are the etiologies of stroke; ; Leukoariosis refers to the grade of leukoencephalopathy calculated by van Swieten scale[^34^](#_ENREF_34) on 24-hours CT; Glomerular filtration rate (GFR) estimated by Cockcroft-Gault formula, Brain Natriuretic Peptide (BNP), C-reactive protein (CRP), Low-density lipoprotein (LDL), High-density lipoprotein (LDL), triglycerides (TG), glycated hemoglobin (HbA1C), arterial blood pressure (BP), cerebrovascular resistance index (CVRi), end-tidal carbon dioxide (EtCO2), low (LF, 0 – 0.15 Hz) and high (HF, 0.15 – 0.5 Hz) frequency spectral bands. For units see table 1.

* P< 0.01 for linear regression analysis, †P< 0.05 after adjustment to age, gender, NIHSS and all independent variables significantly associated with the TFA parameter in univariate analysis.
